# Editorial: Applications of biological networks in biomedicine

**DOI:** 10.3389/fmolb.2022.1005183

**Published:** 2023-01-10

**Authors:** Vinicius Maracaja-Coutinho, Alex Di Genova, Anne Siegel, Mauricio Latorre

**Affiliations:** ^1^ Systemix, Proyecto Anillo ACT210004, Rancagua, Chile; ^2^ Advanced Center for Chronic Diseases – ACCDiS, Facultad de Ciencias Químicas y Farmacéuticas, Universidad de Chile, Santiago, Chile; ^3^ DiGenoma Lab, Instituto de Ciencias de la Ingeniería, Universidad de O’Higgins, Rancagua, Chile; ^4^ Université de Rennes, Inria, CNRS, IRISA, Rennes, France; ^5^ Laboratorio de Bioinformática y Expresión Génica, INTA, Universidad de Chile, Santiago, Chile; ^6^ Laboratorio de Bioingeniería, Instituto de Ciencias de la Ingeniería, Universidad de O’Higgins, Rancagua, Chile

**Keywords:** systems biology, biological networks, biomedicine, mathematical modeling, OMICs technics

The field of biomedicine has required the development of Systems Biology studies to understand and analyze phenomena in an integral way. The aim of this special is to exert a real impact on clinical practice and medicine in different areas, such as disease control, early identification of diseases, and development of biomarkers, among others. In this regard, the constant growth of information on a global scale has led to the development of different bioinformatics strategies, which allow the integration of large amounts of data.

Taking this into consideration, the main objective of this Research Topic, entitled “*Applications of Biological Networks in Biomedicine*”, was to present a series of articles describing the development or use of different platforms on a global scale. Twenty-four different authors managed to deliver valuable information in the fields of metabolomics, multimorbidity, folliculogenesis, and the development of methods to study pathogens clinically resistant to multiple drugs, all of which are relevant biomedicine topics.

First, Ganesan et al. used 1H nuclear magnetic resonance (1H-NMR) to investigate the metabolic effects of single-walled carbon nanotubes (SWCNT) on zebrafish. The analysis of the metabolomics profiling provided a global perspective of the global impact of SWCNT on different metabolic pathways, highlighting those metabolites associated with energy production, amino acids, and nucleotides biosynthesis. These important findings revealed the effects of exposure to organic molecules.

Folliculogenesis is the development of the female germ cell within the somatic cells of the ovary, which matures into a fertilizable egg. Bernabò et al. used a computational biology approach to identify new metabolic sensor molecules that controlled this process and were related to functional endpoints, such as the FSH receptor and steroidogenesis. These results served as a basis for designing innovative diagnostic and treatment methods to preserve female fertility.

Multimorbidity can be defined as the simultaneous presence of two or more chronic diseases. In this context, Dash et al. presented an extensive description of the potential of metabolomics to gain insight into multimorbidity, and also discussed the role of gut microbiota in this pathology. This knowledge led to the development of new treatments related to prebiotics, probiotics, and symbiotic supplementation.

Finally, Tao et al. developed a new test to evaluate certain metabolites that enable antibiotics to kill bacterial pathogens (known as minimum killing concentration, MKC); this interesting approach accelerated the identification of compounds that promote antibiotic-mediated killing efficacy.

As the editor team, we thank and appreciate all authors for their excellent work and commitment to this special issue. We truly believe that our aim was clearly fulfilled because essential findings in Systems Biology were brought together in the context of Biomedicine.

